# Moods in Clinical Depression Are More Unstable than Severe Normal Sadness

**DOI:** 10.3389/fpsyt.2017.00056

**Published:** 2017-04-12

**Authors:** Rudy Bowen, Evyn Peters, Steven Marwaha, Marilyn Baetz, Lloyd Balbuena

**Affiliations:** ^1^University of Saskatchewan, Saskatoon, SK, Canada; ^2^Division of Mental Health and Wellbeing, Warwick Medical School, Warwick University, Coventry, UK; ^3^Affective Disorder Service (IPU 3-8), Caludon Centre, Coventry, UK

**Keywords:** depression, distress, low mood, high mood, mood instability

## Abstract

**Objective:**

Current descriptions in psychiatry and psychology suggest that depressed mood in clinical depression is similar to mild sadness experienced in everyday life, but more intense and persistent. We evaluated this concept using measures of average mood and mood instability (MI).

**Method:**

We prospectively measured low and high moods using separate visual analog scales twice a day for seven consecutive days in 137 participants from four published studies. Participants were divided into a non-depressed group with a Beck Depression Inventory score of ≤10 (*n* = 59) and a depressed group with a Beck Depression Inventory score of ≥18 (*n* = 78). MI was determined by the mean square successive difference statistic.

**Results:**

Mean low and high moods were not correlated in the non-depressed group but were strongly positively correlated in the depressed group. This difference between correlations was significant. Low MI and high MI were weakly positively correlated in the non-depressed group and strongly positively correlated in the depressed group. This difference in correlations was also significant.

**Conclusion:**

The results show that low and high moods, and low and high MI, are highly correlated in people with depression compared with those who are not depressed. Current psychiatric practice does not assess or treat MI or brief high mood episodes in patients with depression. New models of mood that also focus on MI will need to be developed to address the pattern of mood disturbance in people with depression.

## Introduction

The purpose of this paper is to distinguish between sadness and depression. Current descriptions suggest that depression is caused mainly by exposure to stress and the ups and downs of life (1, 2). If depression were simply extreme sadness, then patients would have more control over symptoms, and it would be less stigmatizing as a consequence (3, 4). Freud provided a clinical foundation for this idea by concluding that mourning and melancholia were comparable because the symptoms are similar, they are both precipitated by loss and improve with time (5). He noted that people with melancholia could become over-talkative and manic but did not adequately explain why this is so (5).

Other influential writers in psychiatry and psychology have endorsed similar notions. Bowlby compared negative emotions in adults to those that occur during separation from the attachment figure in infants (6). Beck attributed depression to dysfunctional negative thoughts of defeat, failure, and rejection (7). He dismissed mood-swings as a normal phenomenon (7). Although Watson paid more attention to mood instability (MI), he also dismissed negative mood variability as mundane and of little importance (8).

Kraepelin was a notable exception in that he emphasized brief moods swings and rapidly alternating mood symptoms in patients with manic–depressive illness (9). Consistent with his work, recent studies have shown that patients with mood disorders report increased affective lability and emotional instability; and this also occurs during euthymic periods in bipolar patients (10–12).

Depression could be understood as a consequence of loss or stress, but if high moods do occur during a depressive episode, they tend to be ignored (2, 6), dismissed (7, 8), or isolated to separate categories with low prevalences of about 1% such as bipolar mood disorder (13) and borderline personality disorder (14). Recently, DSM-5 has introduced the specifier “with mixed features” to acknowledge manic/hypomanic symptoms, but three out of seven symptoms are required (13). Any attempt to expand the bipolar spectrum to account for brief periods of high mood (15–17) has been vigorously criticized (18–21).

The reliance on retrospective methods for clinical and research interviewing (22) has made it easier to dismiss brief high moods because retrospective recall in depressed people is biased toward the negative. Recalling mood over the previous 2 weeks is more likely to result in smoothing away mood variation (23–25). If so, the raw data for clinically diagnosing major depression are systematically distorted (26). Ecological momentary assessment, which asks patients to record affect at a given moment over a specified duration, provides a fuller picture of mood by capturing variation in addition to severity. As early as 2006, the US Food and Drug Administration recommended that pharmaceutical companies make use of real-time data instead of patient recollection (26). Increasingly, smartphones are being used as a tool for ecological momentary assessment in psychological and clinical studies (27, 28).

Another way by which recalled moods are distorted is the “common-sense” view that low mood and high mood are mutually exclusive or negatively correlated (8, 29). Since negative affect predominates in depressed people, brief episodes of positive affect tend to be subsumed under overall gloom in patient recollections. There is evidence, however, that positive and negative affect are independent of each other (30) and that people can feel both happy and sad at the same time (31).

Among clinical samples, when low and high moods are measured prospectively on separate axes, the rapid cyclic recurrence of high moods with low moods becomes apparent, a phenomenon known as mood instability (11, 32–34). MI has been shown to exist in up to 13.9% of the adult population (34). MI seems to be the essential component of neuroticism (35, 36) and is an antecedent to major depression (37), psychotic symptoms (38), severity of distress (39), suicidal thoughts (35), and self-harm behavior (40). In this study, we investigated how MI might characterize the experience of people who were depressed as compared with those who were not depressed.

### Hypothesis

People who are depressed (distressed with negative mood and symptoms) are more likely to have more strongly correlated low and high unstable moods than people who are not depressed.

## Materials and Methods

### Participants

We used data from participants in four controlled studies (*n* = 168) that we have published (32, 41–43). All patients had been referred by family physicians for treatment and were all under treatment at the time of the study. In three of the studies, males and females had been referred to general outpatient practices, in one study women had been referred for alcohol abuse, but all had been alcohol free for 3 weeks. All patients completed mood diaries (described in the next section) over a week. All of the studies received approval from the university ethics board. The patient group (*n* = 104) was assessed with the Mini-International Neuropsychiatric Interview (MINI English Version 5.0.0 for DSM-IV), and 49 met criteria for major depression (44). There was high comorbidity with anxiety disorders. Twenty-two of those with major depression (45%) reported hyperthymic symptoms in the past, which did not meet criteria for hypomania (45). The controls (*n* = 64) included health-care personnel and 17 graduate students.

### Procedure

Participants completed the Beck Depression Inventory-IA (46, 47), which is a 21-item retrospective self-report questionnaire. Statements are presented in the first-person (“I feel sad”), and subjects select the item that best reflects their recent state from a choice of four items. It is reliable and correlates well with other measures of depression but may in part assess a general distress or neuroticism factor (48–50). In a study with undergraduate students, the BDI showed strong latent dimensional structure that is there was no evidence that a cut-score defined a latent class of depression (50).

Participants completed separate visual analog scales (VAS) (8) for low mood and high mood. They rated their moods in the morning after awakening and at night before bedtime, for 7 consecutive days (41, 51). The anchor points were “not at all” and “very much so.”

For clarity, we define “low mood” as participant ratings for “sad/blue” or “depressed” over 1 week (twice daily ratings for a total of 14 ratings). Similarly, “high mood” is defined as participant ratings for “enthusiastic/interested” or “high mood.” “Low MI” refers to the fluctuation of low mood, and “high MI” refers to the fluctuation of high mood over the same period. MI is operationalized as the mean square successive difference (MSSD) statistic across 14 ratings (52). One can think of MSSD as the SD of ratings, taking temporal sequence into account.

### Analysis

We used the BDI scores to divide the participants into two distinct groups: those who were within a “normal” range of BDI scores (BDI ≤10) (*n* = 59) (47) and those with BDI scores ≥18 or who were likely clinically depressed (*n* = 78) (46, 47). The remaining participants (*n* = 31) fell outside of these two groups and were excluded from the sample. We used the conventional terms “non-depressed” (BDI ≤10) and “depressed” (≥18) to designate the two groups without assuming that the cut-score of BDI ≥18 defined a latent class of major depression or any particular form of depression. The mean BDI scores of the non-depressed and depressed groups were 4.51 (SD: 2.59) and 20.05 (SD: 13.89), respectively.

We used Pearson’s correlation as a measure of association between low and high moods. We then tested whether the correlations between (a) mean low and high mood and (b) low and high MI were different between the non-depressed and depressed groups. This comparison of correlation magnitudes is, in principle, similar to Meehl’s MAXCOV procedure in which the correlation of two variables is compared along successive cuts in a third variable (22).

## Results

In the original group of referred patient participants (*N* = 104), mean low mood was correlated with low MI; as were mean high mood and high MI (*p* < 0.001). In the original group of controls (*N* = 64), mean low mood was correlated with low MI (*p* < 0.001); as were mean high mood and high MI (*p* < 0.01).

### Sample and Group Demographics

The participants (*n* = 137) ranged in age from 15 to 64 years (mean age = 30.0 years, SD = 10.9), and 104 (75.9%) were females. The “depressed” group (*n* = 78) (mean age = 30.62, SD = 11.60; 80.8% females) was composed of 51 patients, 24 volunteers as “controls” in the original studies, and 3 graduate students. The “non-depressed group” (*n* = 59) (mean age = 29.15 years, SD = 9.66; 69.5% females) included 40 people who volunteered as controls from the original studies, 14 graduate students, and 5 people referred as patients. There was no difference in age or sex distribution between the two groups.

### Mean Mood

Table 1 shows mood scores for both groups. Compared to the non-depressed group, the depressed group experienced more severe low mood (*t* = −4.73, df = 129, *p* < 0.001) and less severe high mood (*t* = 5.41, df = 135, *p* < 0.001), consistent with the selection into non-depressed and depressed groups. Table 2 shows mean low and mean high mood correlations for both groups. Notably, in the non-depressed group, the correlation between mean low and mean high moods was not significant (*r* = −0.01), but in the depressed group, mean low and high moods were positively correlated (*r* = 0.38). The Fisher *r*-to-*z* transformation indicated that the difference between these correlations was significant (*z* = −2.30, *p* = 0.02, two-tailed).

**Table 1:** **Mood and mood instability (MI) in depressed and non-depressed groups**.

	Non-depressed			Depressed			*t*	df	Sig (two-tailed)

	*N*	Mean	SD	*N*	Mean	SD	–	–	–
Low mood	59	1.50	1.34	78	2.95	2.22	−4.73	129.22	<0.00
High mood	59	4.81	1.69	78	3.05	2.13	5.41	134.68	<0.00
Low MI	59	1.72	1.30	78	2.40	1.49	−2.81	135	<0.01
High MI	59	2.84	1.06	78	2.44	1.60	1.77	132.94	0.08

*Low, high mood: mean visual analog scale mood ratings for low and high moods*.

*Low, high MI: mean square successive difference statistic for low mood and high mood*.

**Table 2:** **Correlations**.^a^

	Non-depressed			Depressed				
	*n*	*r*^0^	95% confidence limits	*n*	*r*	95% confidence limits	*Z*	Sig of the difference in *r*^0^ and *r* (two-tailed)
Correlation mean low mood and high mood	59	−0.01	−0.27–0.25	78	0.38^b^	0.17–0.55	−2.3	0.02
Correlation mean square successive difference (MSSD) (mood instability) low mood and MSSD high mood	59	0.31^a^	0.06–0.53	78	0.61^a^	0.45–0.73	−2.18	0.03

*^a^Correlations between mean low and mean high moods for the non-depressed and depressed groups and MSSD low and MSSD high moods for the non-depressed and depressed groups*.

*^b^Correlation is significant at the 0.05 level (two-tailed)*.

### Mood Instability

The depressed group experienced more severe low MI than the non-depressed group, as consistent with the findings of the original individual studies. The difference in high MI between the depressed and non-depressed groups was not significant. Table 2 shows the important finding that low MI and high MI were correlated in both the depressed and the non-depressed groups, but the magnitude of correlation for the depressed group (*r* = 0.61) was almost twice that for the non-depressed group (*r* = 0.31). The difference between these correlations was significant (*z* = −2.18, *p* = 0.03, two-tailed).

## Discussion

On the VAS ratings, the depressed group experienced more severe low moods and less severe high moods than the non-depressed group, as would be expected given the selection criteria. This is consistent with reports of more severe negative emotions and variable positive emotions in ecological momentary assessment studies of patients with major depression (12, 33, 53).

In the non-depressed group, the overall means of low mood and high mood were uncorrelated. This supports the observation that in normal people low and high moods are not strongly related and easily distinguished (8). In the depressed group, however, mean low mood and mean high mood were moderately positively correlated. This indicates that the depressed group experienced high moods concurrent with low moods (54, 55). This is contrary to the common-sense view that low mood should not be associated with high moods.

Low MI and high MI were weakly correlated in the non-depressed group. In the depressed group, the correlation was moderate to large, and the difference between these correlations was significant. In other words, in the depressed group, the fluctuations of low moods and high moods are more closely related (Table 2).

Taken together, these results suggest that in people with depression, mood is a complex combination of rapidly fluctuating seemingly polar opposite emotions. This distinction is more easily understood if MI is considered along with stable low mood (56). Other studies have shown complex emotional patterns in anxiety and mood disorders (12, 56–58). Two clinical applications are (a) that the usual semi-structured retrospective assessment might provide a limited appreciation of “nuanced” mood symptoms (12) and (b) that attention to mood stabilization might add an extra dimension to treatment (12, 59). In other words, since MI reflects neuroticism (36) that is an antecedent of depression (60), attention to the assessment and treatment of MI in addition to specific symptoms of depression (61, 62) might increase the treatment efficacy.

Our study had several methodological limitations. First, the number of participants was relatively small and from one center. Second, the wording of the questions for low mood and high mood varied slightly between studies, although the words were similar. This might be an advantage by reflecting real-world interviewing conditions. Third, paper and pencil diaries were used, raising the possibility of people retrospectively filling in data. Participants understood that they were to record momentary mood ratings, and the method of calculating MI was not intuitively apparent to the participant (52). Furthermore, all of the individual study results produced clear differences suggesting that the participants understood the instructions. Fourth, choosing graduate students as controls in one of the studies was a convenience sample, but we considered that graduate students would be generally more stable than undergraduates and that they would be older and closer in age to patients. Fifth, for simplicity we considered only low and high moods. Moods in broadly defined depression would likely appear even more complex and distressing if anxiety, irritability, and psychotic symptoms were included (63). Sixth, five people in the “non-depressed” group had been referred as patients. These people may have improved while waiting for treatment or may have had symptoms that were not detected by the way that they completed the BDI. Finally, during the diagnostic interview we did not probe sufficiently for hypomanic symptoms during the depressive episode, so we cannot say how many of the participants would have met criteria for the DSM-5 category of major depression with mixed features (13).

There are also limitations to the conclusions that can be drawn. The correlation between low and high MI might not be the best representation of complex emotions. The data do not address the question of the relative merits of continuous or categorical approaches to classification in psychiatry. Recent taxometric analyses indicate that in the mood and anxiety domains, dimensional distributions are much more likely (64, 65). We studied MI as a phenomenon, similar to instability in physical parameters such as blood pressure (66) and blood sugar (67) and without assumptions as to cause. MI is considered to be a simpler concept than “affective instability due to a marked reactivity of mood” (13) or emotional dysregulation (68). One question for further studies would be whether MI leads to unstable interpersonal relationships or *vice versa*. The relationship of MI to interpersonal and social–environmental events is a matter for further research. Instability of low moods is not entirely accounted for by reactivity to negative events (12). Finally, associating MI with neuroticism does not detract from evidence that certain kinds of stress can affect depression (69, 70). The main advantage to this study is the longitudinal collection of data, which is likely to give a more accurate depiction of moods than retrospective studies (23).

The results show that low and high moods, and low and high MI, are highly correlated in people with depression compared with those who are not depressed. Current psychiatric practice does not assess or treat MI or brief high mood episodes in patients with depression. New models of mood that also focus on MI will need to be developed to address the pattern of mood disturbance in people with depression.

## Ethics Statement

This study was carried out in accordance with the recommendations of the Canadian Tri-Council Policy Statement for Ethical Conduct in research involving humans with written informed consent from all subjects. All subjects gave written informed consent in accordance with the Declaration of Helsinki. The protocol was approved by the University of Saskatchewan Behavioural Ethics Board.

## Author Contributions

RB: study conception, drafting the article, revised the article after peer review, and responded to reviewer comments. EP: contributing to the concepts as well as reading and adding to the final version. SM and MB: contributing to the concepts as well as reading and providing approval to the final version. LB: conception of the work, analysis of the data, and revised the article after peer review.

## Conflict of Interest Statement

The authors declare that the research was conducted in the absence of any commercial or financial relationships that could be construed as a potential conflict of interest.
